# Bis(1,10-phenanthroline-κ^2^ *N*,*N*′)(sulfato-κ^2^ *O*,*O*′)cadmium(II) propane-1,3-diol solvate

**DOI:** 10.1107/S1600536810022518

**Published:** 2010-06-18

**Authors:** Kai-Long Zhong, Jiang-Dong Cui

**Affiliations:** a Department of Applied Chemistry, Nanjing College of Chemical Technology, Nanjing 210048, People’s Republic of China

## Abstract

In the title compound, [Cd(SO_4_)(C_12_H_8_N_2_)_2_]·C_3_H_8_O_2_, the Cd^II^ atom has a distorted octa­hedral coordination composed of four N atoms from two chelating 1,10-phenanthroline ligands and two O atoms from an *O*,*O*′-bidentate sulfate group. The two chelating NCCN groups subtend a dihedral angle of 82.21 (9)°. The Cd^II^ ion, the S atom and the middle C atom of the propane-1,3-diol solvent mol­ecule are located on special positions, site symmetry 2. The solvate features a pair of O—H⋯O hydrogen bonds with the uncoordinated O atoms of the sulfate ion. The OH group of the propane-1,3-diol solvent is disordered over two positions of equal occupancy.

## Related literature

For isostructural compounds, see: Cui *et al.* (2010 ▶); Ni *et al.* (2010 ▶); Zhong (2010*a* ▶). For the ethane-1,2-diol solvate of the title complex, see: Lu *et al.* (2006 ▶). For background to bidentate-chelating sulfate complexes, see: Zhong *et al.* (2006 ▶, 2010*b* ▶); Zhu *et al.* (2006 ▶). For the preparation, see: Zhong *et al.* (2010*a* ▶). For background to coordination polymers, see: Batten & Robson (1998 ▶); Eddaoudi *et al.* (2001 ▶); Li *et al.* (2003 ▶).
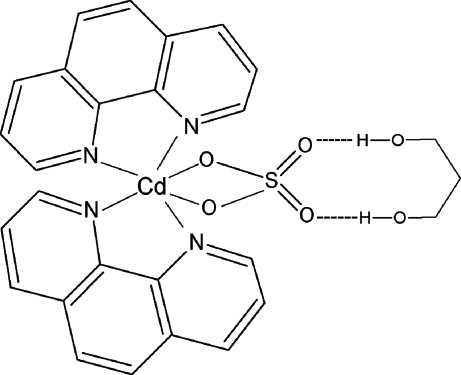


## Experimental

### 

#### Crystal data

[Cd(SO_4_)(C_12_H_8_N_2_)_2_]·C_3_H_8_O_2_
*M* *_r_* = 644.98
Monoclinic, 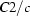

*a* = 17.854 (4) Å
*b* = 12.520 (3) Å
*c* = 13.519 (3) Å
β = 123.01 (3)°
*V* = 2534.1 (13) Å^3^
*Z* = 4
Mo *K*α radiation
μ = 1.00 mm^−1^
*T* = 223 K
0.40 × 0.30 × 0.20 mm

#### Data collection

Rigaku Mercury CCD diffractometer
Absorption correction: multi-scan (*REQAB*; Jacobson, 1998 ▶) *T*_min_ = 0.691, *T*_max_ = 0.826
8349 measured reflections
2880 independent reflections
2683 reflections with *I* > 2σ(*I*)
*R*_int_ = 0.021

#### Refinement

*R*[*F* ^2^ > 2σ(*F* ^2^)] = 0.028
*wR*(*F* ^2^) = 0.072
*S* = 1.10
2880 reflections
178 parameters
3 restraints
H-atom parameters constrained
Δρ_max_ = 0.75 e Å^−3^
Δρ_min_ = −0.65 e Å^−3^

### 

Data collection: *CrystalClear* (Rigaku, 2007 ▶); cell refinement: *CrystalClear*; data reduction: *CrystalClear*; program(s) used to solve structure: *SHELXS97* (Sheldrick, 2008 ▶); program(s) used to refine structure: *SHELXL97* (Sheldrick, 2008 ▶); molecular graphics: *XP* in *SHELXTL* (Sheldrick, 2008 ▶); software used to prepare material for publication: *SHELXTL*.

## Supplementary Material

Crystal structure: contains datablocks global, I. DOI: 10.1107/S1600536810022518/bq2222sup1.cif

Structure factors: contains datablocks I. DOI: 10.1107/S1600536810022518/bq2222Isup2.hkl

Additional supplementary materials:  crystallographic information; 3D view; checkCIF report

## Figures and Tables

**Table 1:** Selected bond lengths (Å)

Cd1—N2	2.3255 (19)
Cd1—N1	2.3439 (19)
Cd1—O1	2.3608 (17)
S1—O2	1.4652 (16)
S1—O1	1.4873 (17)

**Table 2:** Hydrogen-bond geometry (Å, °)

*D*—H⋯*A*	*D*—H	H⋯*A*	*D*⋯*A*	*D*—H⋯*A*
O3—H3*B*⋯O2	0.82	2.05	2.806 (3)	153

**Fig. 1.**
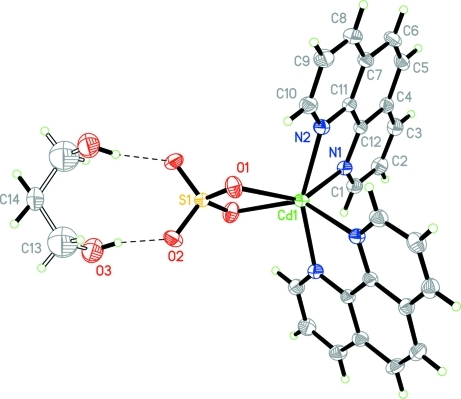
The molecular structure showing the atom-numbering scheme with displacement ellipsoids drawn at the 50% probability level. The dashed lines represent O—H···O interactions. Unlabeled atoms are related to the labeled atoms by the symmetry operator(-x + 1, y, - z + 1/2).

**Fig. 2.**
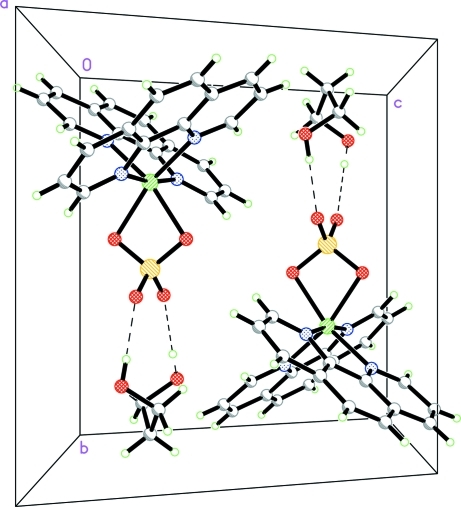
Packing diagram of the title compound.

## supplementary crystallographic information

### Comment

The design and synthesis of new coordination polymers have attracted great attention in recent years, owing to their interesting structural topologies and potential application as functional materials (Batten & Robson,1998; Eddaoudi *et al.*, 2001; Li *et al.*, 2003). Four years ago, we attempted to synthesize mixed-ligand coordination polymers of transition metal with phen as second ligand *via* a ethanediol-solvothermal reaction, unexpectedly, we found the potentially interesting structure with bidentate-chelating sulfate ligand, *e.g.* [CdSO_4_(C_12_H_8_N_2_)_2_].C_2_H_6_O_2_, (II) (C_12_H_8_N_2_ is 1,10- phenanthroline; Lu *et al.*, 2006), [CoSO_4_(C_12_H_8_N_2_)_2_].C_2_H_6_O_2_, (III) (Zhong *et al.*,, 2006), [ZnSO_4_(C_12_H_8_N_2_)_2_].C_2_H_6_O_2_, (IV) (Zhu *et al.*, 2006). We report here the structure of [CdSO_4_(C_12_H_8_N_2_)_2_].C_2_H_6_O_2_, (I).

X-ray diffraction indicated that the title compound, (I) is isostructural to the recently reported cobalt(II), nickel(II) and zinc(II) structure with bidentate-chelating sulfate ligand (Zhong, 2010; Cui *et al.*, 2010; Ni *et al.*, 2010). The geometry of the phen and sulfate ligands is in good agreement with those reported in the three isomorphs complexes. The Cd^II^ metal ions has an octahedral coordination environment, with four N atoms from two phen ligands and two O atoms from a O,*O*'-bidentate sulfate group. The Zn^II^ ion, S atom and the mid-carbon atom of the propane-1,3-diol solvent molecule lie on a special position of site symmetry 2 [symmetry code: -*x* + 1, *y*, - *z* + 1/2]. The dihedral angle (82.2°) between the two chelating NCCN groups are larger than that found in (II) [74.5°; Lu *et al.*, 2006]. The Cd—N bond distance [2.3258 (19)–2.3441 (19) Å], the N—Cd—N bite angle [72.00 (7)°], the O—Cd—O bite angle [60.39 (8)°] and the Cd—O bond distance [2.3605 (17) Å] are are in good accord with those found in the (II) [71.91 (7)°, 2.327 (2)–2.343 (2) Å, 59.98 (9)° and 2.361 (2) Å, respectively]. Selected coordination bond distances and angles in Table 1. In the crystal structure, a pair of intermolecular O—H···O hydrogen bonds help to further stabilize structure (see Fig. 1 and Table 2).

Fig. 2 shows the crystal packing of the title compound. The molecular twofold axis is along the direction of the molecular dipole moment and the complexes are packed with their dipole moments alternately along the *b* axis directions.

### Experimental

Colorless block-shaped crystal of the title compound was obtained by the similar route that described by Zhong (2010*a*), with ZnSO_4_.7H_2_O in place of NiSO_4_.7H_2_O

### Refinement

All non-hydrogen atoms were refined anisotropically. All H atoms were placed in geometrically idealized positions and refined as riding atoms, with C—H = 0.97 Å and O—H = 0.82 Å; *U*_iso_(H) = 1.2*U*_eq_(C) and 1.5*U*_eq_(O).

The central carbon of propane-1,3-diol solvent is disordered over two positions with site-occupancy factors of 1/2, sharing a common atom O3. The C13—O3 and C13'—O3 distances were restrained to 1.381 (5)Å and 1.387 (6) Å, respectively.

### Crystal data

**Table:**

[Cd(SO_4_)(C_12_H_8_N_2_)_2_]·C_3_H_8_O_2_	*F*(000) = 1304
*M**_r_* = 644.98	*D*_x_ = 1.691 Mg m^−^^3^
Monoclinic, *C*2/*c*	Mo *K*α radiation, λ = 0.71073 Å
Hall symbol: -C 2yc	Cell parameters from 3776 reflections
*a* = 17.854 (4) Å	θ = 3.1–27.5°
*b* = 12.520 (3) Å	µ = 1.00 mm^−^^1^
*c* = 13.519 (3) Å	*T* = 223 K
β = 123.01 (3)°	Block, colorless
*V* = 2534.1 (13) Å^3^	0.40 × 0.30 × 0.20 mm
*Z* = 4	

### Data collection

**Table:**

Rigaku Mercury CCD diffractometer	2880 independent reflections
Radiation source: fine-focus sealed tube	2683 reflections with *I* > 2σ(*I*)
graphite	*R*_int_ = 0.021
Detector resolution: 28.5714 pixels mm^-1^	θ_max_ = 27.5°, θ_min_ = 3.1°
ω scans	*h* = −19→23
Absorption correction: multi-scan (REQAB; Jacobson, 1998)	*k* = −12→16
*T*_min_ = 0.691, *T*_max_ = 0.826	*l* = −17→12
8349 measured reflections	

### Refinement

**Table:**

Refinement on *F*^2^	Secondary atom site location: difference Fourier map
Least-squares matrix: full	Hydrogen site location: inferred from neighbouring sites
*R*[*F*^2^ > 2σ(*F*^2^)] = 0.028	H-atom parameters constrained
*wR*(*F*^2^) = 0.072	*w* = 1/[σ^2^(*F*_o_^2^) + (0.0391*P*)^2^ + 2.1837*P*] where *P* = (*F*_o_^2^ + 2*F*_c_^2^)/3
*S* = 1.10	(Δ/σ)_max_ < 0.001
2880 reflections	Δρ_max_ = 0.75 e Å^−^^3^
178 parameters	Δρ_min_ = −0.65 e Å^−^^3^
3 restraints	Extinction correction: *SHELXL97* (Sheldrick, 2008), Fc^*^=kFc[1+0.001xFc^2^λ^3^/sin(2θ)]^-1/4^
Primary atom site location: structure-invariant direct methods	Extinction coefficient: 0.0053 (3)

### Special details

**Table:**

Geometry. All e.s.d.'s (except the e.s.d. in the dihedral angle between two l.s. planes) are estimated using the full covariance matrix. The cell e.s.d.'s are taken into account individually in the estimation of e.s.d.'s in distances, angles and torsion angles; correlations between e.s.d.'s in cell parameters are only used when they are defined by crystal symmetry. An approximate (isotropic) treatment of cell e.s.d.'s is used for estimating e.s.d.'s involving l.s. planes.
Refinement. Refinement of *F*^2^ against ALL reflections. The weighted *R*-factor *wR* and goodness of fit *S* are based on *F*^2^, conventional *R*-factors *R* are based on *F*, with *F* set to zero for negative *F*^2^. The threshold expression of *F*^2^ > σ(*F*^2^) is used only for calculating *R*-factors(gt) *etc*. and is not relevant to the choice of reflections for refinement. *R*-factors based on *F*^2^ are statistically about twice as large as those based on *F*, and *R*- factors based on ALL data will be even larger.

### Fractional atomic coordinates and isotropic or equivalent isotropic displacement parameters (Å^2^)

**Table:**

	*x*	*y*	*z*	*U*_iso_*/*U*_eq_	Occ. (<1)
Cd1	0.5000	0.174163 (16)	0.2500	0.02229 (10)	
S1	0.5000	−0.06039 (5)	0.2500	0.01961 (16)	
O1	0.51467 (11)	0.01117 (13)	0.34685 (14)	0.0300 (3)	
O2	0.57895 (10)	−0.12744 (13)	0.29112 (15)	0.0306 (4)	
N1	0.39140 (11)	0.29051 (15)	0.10875 (16)	0.0222 (4)	
N2	0.40091 (11)	0.21462 (15)	0.30577 (17)	0.0234 (4)	
C7	0.27252 (14)	0.31996 (16)	0.2610 (2)	0.0237 (4)	
C9	0.34479 (16)	0.2062 (2)	0.4312 (2)	0.0306 (5)	
H9A	0.3498	0.1780	0.4982	0.037*	
C2	0.32079 (15)	0.39930 (19)	−0.0655 (2)	0.0295 (5)	
H2A	0.3194	0.4232	−0.1316	0.035*	
C8	0.27880 (15)	0.27765 (19)	0.3615 (2)	0.0290 (5)	
H8A	0.2381	0.2983	0.3806	0.035*	
C10	0.40478 (16)	0.17602 (17)	0.4000 (2)	0.0275 (5)	
H10A	0.4493	0.1268	0.4472	0.033*	
C6	0.20366 (14)	0.39348 (18)	0.1827 (2)	0.0282 (5)	
H6A	0.1619	0.4159	0.1991	0.034*	
C11	0.33584 (13)	0.28613 (16)	0.23634 (19)	0.0207 (4)	
C5	0.19881 (15)	0.43057 (17)	0.0856 (2)	0.0272 (5)	
H5A	0.1534	0.4777	0.0357	0.033*	
C4	0.26246 (13)	0.39846 (17)	0.05810 (19)	0.0232 (4)	
C3	0.25909 (15)	0.43470 (18)	−0.0429 (2)	0.0283 (5)	
H3A	0.2150	0.4826	−0.0941	0.034*	
C1	0.38597 (16)	0.32669 (17)	0.0124 (2)	0.0268 (5)	
H1A	0.4274	0.3025	−0.0039	0.032*	
C12	0.33059 (14)	0.32544 (15)	0.13234 (19)	0.0204 (4)	
C14	0.5000	−0.4518 (3)	0.2500	0.0452 (10)	
O3	0.55944 (17)	−0.32203 (16)	0.1763 (2)	0.0548 (6)	
H3B	0.5502	−0.2607	0.1883	0.082*	
C13'	0.5787 (6)	−0.3855 (7)	0.2714 (7)	0.084 (2)*	0.50
H13A	0.5986	−0.3401	0.3396	0.101*	0.50
H13B	0.6276	−0.4330	0.2897	0.101*	0.50
C13	0.4872 (3)	−0.3854 (4)	0.1485 (4)	0.0295 (10)*	0.50
H13E	0.4749	−0.4329	0.0846	0.035*	0.50
H13C	0.4354	−0.3400	0.1201	0.035*	0.50
H14A	0.4484	−0.4976	0.2216	0.035*	

### Atomic displacement parameters (Å^2^)

**Table:**

	*U*^11^	*U*^22^	*U*^33^	*U*^12^	*U*^13^	*U*^23^
Cd1	0.01994 (13)	0.02154 (14)	0.02924 (15)	0.000	0.01589 (10)	0.000
S1	0.0176 (3)	0.0208 (4)	0.0207 (4)	0.000	0.0106 (3)	0.000
O1	0.0381 (9)	0.0256 (7)	0.0242 (8)	0.0034 (7)	0.0156 (7)	−0.0016 (6)
O2	0.0249 (8)	0.0319 (9)	0.0366 (9)	0.0073 (6)	0.0178 (7)	0.0025 (7)
N1	0.0218 (9)	0.0223 (9)	0.0254 (9)	−0.0008 (7)	0.0147 (7)	−0.0011 (7)
N2	0.0227 (9)	0.0231 (9)	0.0274 (10)	0.0011 (7)	0.0155 (8)	0.0017 (7)
C7	0.0225 (10)	0.0246 (11)	0.0262 (11)	−0.0013 (7)	0.0147 (9)	−0.0048 (9)
C9	0.0363 (12)	0.0335 (12)	0.0301 (12)	0.0001 (10)	0.0234 (11)	0.0029 (10)
C2	0.0349 (12)	0.0303 (12)	0.0250 (11)	−0.0036 (10)	0.0174 (10)	0.0012 (10)
C8	0.0297 (11)	0.0317 (12)	0.0341 (13)	0.0002 (9)	0.0229 (10)	−0.0019 (10)
C10	0.0273 (11)	0.0278 (12)	0.0304 (12)	0.0026 (8)	0.0177 (10)	0.0051 (9)
C6	0.0236 (10)	0.0301 (12)	0.0332 (12)	0.0040 (8)	0.0170 (9)	−0.0054 (10)
C11	0.0198 (9)	0.0191 (10)	0.0247 (11)	−0.0017 (7)	0.0131 (8)	−0.0027 (8)
C5	0.0235 (10)	0.0252 (11)	0.0292 (12)	0.0045 (8)	0.0119 (9)	−0.0017 (9)
C4	0.0224 (10)	0.0204 (10)	0.0243 (10)	−0.0011 (8)	0.0112 (8)	−0.0030 (8)
C3	0.0271 (11)	0.0266 (12)	0.0257 (11)	0.0009 (8)	0.0107 (9)	0.0016 (9)
C1	0.0293 (11)	0.0276 (12)	0.0293 (12)	−0.0022 (8)	0.0196 (10)	−0.0023 (9)
C12	0.0189 (9)	0.0192 (10)	0.0231 (10)	−0.0022 (7)	0.0115 (8)	−0.0029 (8)
C14	0.050 (2)	0.0260 (19)	0.060 (3)	0.000	0.030 (2)	0.000
O3	0.0824 (17)	0.0422 (12)	0.0729 (16)	−0.0064 (10)	0.0636 (15)	−0.0086 (10)

### Geometric parameters (Å, °)

**Table:**

Cd1—N2^i^	2.3255 (19)	C8—H8A	0.9300
Cd1—N2	2.3255 (19)	C10—H10A	0.9300
Cd1—N1^i^	2.344 (2)	C6—C5	1.351 (3)
Cd1—N1	2.3439 (19)	C6—H6A	0.9300
Cd1—O1^i^	2.3608 (17)	C11—C12	1.444 (3)
Cd1—O1	2.3608 (17)	C5—C4	1.432 (3)
Cd1—S1	2.9366 (10)	C5—H5A	0.9300
S1—O2^i^	1.4652 (16)	C4—C3	1.409 (3)
S1—O2	1.4652 (16)	C4—C12	1.412 (3)
S1—O1	1.4873 (17)	C3—H3A	0.9300
S1—O1^i^	1.4873 (17)	C1—H1A	0.9300
N1—C1	1.332 (3)	C14—C13^i^	1.512 (5)
N1—C12	1.360 (3)	C14—C13	1.512 (5)
N2—C10	1.328 (3)	C14—C13'	1.518 (9)
N2—C11	1.358 (3)	C14—C13'^i^	1.518 (9)
C7—C8	1.405 (3)	C14—H14A	0.9699
C7—C11	1.407 (3)	O3—C13	1.380 (5)
C7—C6	1.436 (3)	O3—C13'	1.385 (7)
C9—C8	1.367 (3)	O3—H3B	0.8200
C9—C10	1.400 (3)	C13'—H13A	0.9700
C9—H9A	0.9300	C13'—H13B	0.9700
C2—C3	1.367 (3)	C13—H13E	0.9700
C2—C1	1.398 (3)	C13—H13C	0.9700
C2—H2A	0.9300		
			
N2^i^—Cd1—N2	154.84 (9)	N2—C10—C9	122.8 (2)
N2^i^—Cd1—N1^i^	72.00 (7)	N2—C10—H10A	118.6
N2—Cd1—N1^i^	92.19 (7)	C9—C10—H10A	118.6
N2^i^—Cd1—N1	92.19 (7)	C5—C6—C7	120.8 (2)
N2—Cd1—N1	72.00 (7)	C5—C6—H6A	119.6
N1^i^—Cd1—N1	103.15 (9)	C7—C6—H6A	119.6
N2^i^—Cd1—O1^i^	83.26 (6)	N2—C11—C7	122.0 (2)
N2—Cd1—O1^i^	119.60 (6)	N2—C11—C12	118.37 (18)
N1^i^—Cd1—O1^i^	141.41 (6)	C7—C11—C12	119.60 (19)
N1—Cd1—O1^i^	107.02 (6)	C6—C5—C4	121.1 (2)
N2^i^—Cd1—O1	119.60 (6)	C6—C5—H5A	119.5
N2—Cd1—O1	83.26 (6)	C4—C5—H5A	119.5
N1^i^—Cd1—O1	107.02 (6)	C3—C4—C12	117.6 (2)
N1—Cd1—O1	141.41 (6)	C3—C4—C5	122.7 (2)
O1^i^—Cd1—O1	60.38 (8)	C12—C4—C5	119.7 (2)
N2^i^—Cd1—S1	102.58 (5)	C2—C3—C4	119.9 (2)
N2—Cd1—S1	102.58 (5)	C2—C3—H3A	120.1
N1^i^—Cd1—S1	128.42 (5)	C4—C3—H3A	120.1
N1—Cd1—S1	128.42 (5)	N1—C1—C2	123.0 (2)
O1^i^—Cd1—S1	30.19 (4)	N1—C1—H1A	118.5
O1—Cd1—S1	30.19 (4)	C2—C1—H1A	118.5
O2^i^—S1—O2	110.10 (14)	N1—C12—C4	122.0 (2)
O2^i^—S1—O1	110.53 (10)	N1—C12—C11	118.83 (18)
O2—S1—O1	109.85 (10)	C4—C12—C11	119.14 (19)
O2^i^—S1—O1^i^	109.85 (10)	C13^i^—C14—C13	113.3 (4)
O2—S1—O1^i^	110.53 (10)	C13^i^—C14—C13'	82.0 (4)
O1—S1—O1^i^	105.92 (14)	C13—C14—C13'	62.5 (4)
O2^i^—S1—Cd1	124.95 (7)	C13^i^—C14—C13'^i^	62.5 (4)
O2—S1—Cd1	124.95 (7)	C13—C14—C13'^i^	82.0 (4)
O1—S1—Cd1	52.96 (7)	C13'—C14—C13'^i^	113.7 (8)
O1^i^—S1—Cd1	52.96 (7)	C13^i^—C14—H14A	109.0
S1—O1—Cd1	96.85 (8)	C13—C14—H14A	109.0
C1—N1—C12	118.56 (19)	C13'—C14—H14A	168.7
C1—N1—Cd1	126.53 (15)	C13'^i^—C14—H14A	70.5
C12—N1—Cd1	114.87 (14)	C13—O3—C13'	69.3 (4)
C10—N2—C11	118.73 (19)	C13—O3—H3B	109.5
C10—N2—Cd1	125.46 (15)	C13'—O3—H3B	109.2
C11—N2—Cd1	115.78 (14)	O3—C13'—C14	113.6 (6)
C8—C7—C11	117.7 (2)	O3—C13'—H13A	108.8
C8—C7—C6	122.7 (2)	C14—C13'—H13A	108.8
C11—C7—C6	119.6 (2)	O3—C13'—H13B	108.8
C8—C9—C10	118.8 (2)	C14—C13'—H13B	108.8
C8—C9—H9A	120.6	H13A—C13'—H13B	107.7
C10—C9—H9A	120.6	O3—C13—C14	114.3 (3)
C3—C2—C1	119.0 (2)	O3—C13—H13E	108.7
C3—C2—H2A	120.5	C14—C13—H13E	108.7
C1—C2—H2A	120.5	O3—C13—H13C	108.7
C9—C8—C7	119.9 (2)	C14—C13—H13C	108.7
C9—C8—H8A	120.1	H13E—C13—H13C	107.6
C7—C8—H8A	120.1		
			
N2^i^—Cd1—S1—O2^i^	−140.99 (10)	N1—Cd1—N2—C11	−3.31 (14)
N2—Cd1—S1—O2^i^	39.01 (10)	O1^i^—Cd1—N2—C11	−103.06 (15)
N1^i^—Cd1—S1—O2^i^	142.14 (10)	O1—Cd1—N2—C11	−153.30 (15)
N1—Cd1—S1—O2^i^	−37.86 (10)	S1—Cd1—N2—C11	−129.96 (14)
O1^i^—Cd1—S1—O2^i^	−89.51 (12)	C10—C9—C8—C7	−0.4 (4)
O1—Cd1—S1—O2^i^	90.49 (12)	C11—C7—C8—C9	−0.1 (3)
N2^i^—Cd1—S1—O2	39.01 (10)	C6—C7—C8—C9	178.6 (2)
N2—Cd1—S1—O2	−140.99 (10)	C11—N2—C10—C9	−0.3 (3)
N1^i^—Cd1—S1—O2	−37.86 (10)	Cd1—N2—C10—C9	177.81 (17)
N1—Cd1—S1—O2	142.14 (10)	C8—C9—C10—N2	0.6 (4)
O1^i^—Cd1—S1—O2	90.49 (12)	C8—C7—C6—C5	−178.6 (2)
O1—Cd1—S1—O2	−89.51 (12)	C11—C7—C6—C5	0.0 (3)
N2^i^—Cd1—S1—O1	128.52 (9)	C10—N2—C11—C7	−0.3 (3)
N2—Cd1—S1—O1	−51.48 (9)	Cd1—N2—C11—C7	−178.50 (15)
N1^i^—Cd1—S1—O1	51.65 (10)	C10—N2—C11—C12	−178.31 (19)
N1—Cd1—S1—O1	−128.35 (10)	Cd1—N2—C11—C12	3.4 (2)
O1^i^—Cd1—S1—O1	180.0	C8—C7—C11—N2	0.4 (3)
N2^i^—Cd1—S1—O1^i^	−51.48 (9)	C6—C7—C11—N2	−178.3 (2)
N2—Cd1—S1—O1^i^	128.52 (9)	C8—C7—C11—C12	178.45 (19)
N1^i^—Cd1—S1—O1^i^	−128.35 (10)	C6—C7—C11—C12	−0.2 (3)
N1—Cd1—S1—O1^i^	51.65 (10)	C7—C6—C5—C4	−0.5 (3)
O1—Cd1—S1—O1^i^	180.0	C6—C5—C4—C3	179.4 (2)
O2^i^—S1—O1—Cd1	−118.93 (9)	C6—C5—C4—C12	1.1 (3)
O2—S1—O1—Cd1	119.38 (9)	C1—C2—C3—C4	0.5 (3)
O1^i^—S1—O1—Cd1	0.0	C12—C4—C3—C2	−0.3 (3)
N2^i^—Cd1—O1—S1	−61.43 (10)	C5—C4—C3—C2	−178.6 (2)
N2—Cd1—O1—S1	129.74 (9)	C12—N1—C1—C2	0.3 (3)
N1^i^—Cd1—O1—S1	−140.02 (8)	Cd1—N1—C1—C2	−177.53 (16)
N1—Cd1—O1—S1	80.06 (12)	C3—C2—C1—N1	−0.5 (3)
O1^i^—Cd1—O1—S1	0.0	C1—N1—C12—C4	0.0 (3)
N2^i^—Cd1—N1—C1	20.70 (18)	Cd1—N1—C12—C4	178.00 (15)
N2—Cd1—N1—C1	−179.26 (19)	C1—N1—C12—C11	179.70 (19)
N1^i^—Cd1—N1—C1	92.72 (18)	Cd1—N1—C12—C11	−2.3 (2)
O1^i^—Cd1—N1—C1	−62.92 (19)	C3—C4—C12—N1	0.1 (3)
O1—Cd1—N1—C1	−126.49 (17)	C5—C4—C12—N1	178.45 (19)
S1—Cd1—N1—C1	−87.28 (18)	C3—C4—C12—C11	−179.68 (19)
N2^i^—Cd1—N1—C12	−157.16 (14)	C5—C4—C12—C11	−1.3 (3)
N2—Cd1—N1—C12	2.88 (14)	N2—C11—C12—N1	−0.8 (3)
N1^i^—Cd1—N1—C12	−85.14 (14)	C7—C11—C12—N1	−178.89 (19)
O1^i^—Cd1—N1—C12	119.22 (14)	N2—C11—C12—C4	178.97 (19)
O1—Cd1—N1—C12	55.65 (18)	C7—C11—C12—C4	0.9 (3)
S1—Cd1—N1—C12	94.86 (14)	C13—O3—C13'—C14	−4.0 (5)
N2^i^—Cd1—N2—C10	−128.08 (18)	C13^i^—C14—C13'—O3	−117.7 (7)
N1^i^—Cd1—N2—C10	−78.30 (19)	C13—C14—C13'—O3	3.9 (5)
N1—Cd1—N2—C10	178.6 (2)	C13'^i^—C14—C13'—O3	−62.5 (5)
O1^i^—Cd1—N2—C10	78.82 (19)	C13'—O3—C13—C14	4.1 (5)
O1—Cd1—N2—C10	28.59 (18)	C13^i^—C14—C13—O3	62.8 (3)
S1—Cd1—N2—C10	51.92 (18)	C13'—C14—C13—O3	−3.9 (5)
N2^i^—Cd1—N2—C11	50.04 (14)	C13'^i^—C14—C13—O3	118.1 (4)
N1^i^—Cd1—N2—C11	99.81 (15)		

Symmetry codes: (i) −*x*+1, *y*, −*z*+1/2.

### Hydrogen-bond geometry (Å, °)

**Table:**

*D*—H···*A*	*D*—H	H···*A*	*D*···*A*	*D*—H···*A*
O3—H3B···O2	0.82	2.05	2.806 (3)	153
